# Alzheimer's patient feedback to complement research using model systems for cognitive aging and dementia

**DOI:** 10.3389/fgene.2014.00269

**Published:** 2014-08-06

**Authors:** Shin Murakami, Alexander “Sandy” Halperin

**Affiliations:** ^1^Department of Basic Sciences, College of Osteopathic Medicine, Touro University-CaliforniaVallejo, CA, USA; ^2^Early-Stage Advisory Group/Alumnus (2012-2013), National Alzheimer's AssociationChicago, IL, USA; ^3^Alzheimer's Project, Inc.Tallahassee, FL, USA

**Keywords:** Alzheimer's disease (AD), age-related memory impairment (AMI), age-associated memory impairment (AAMI), *C. elegans*, cognitive impairment, dementia, outreach, stigma

This opinion article was co-authored with an early-stage Alzheimer's patient to assemble essential ideas regarding potential outreach initiatives and to describe the nature of research to and from model systems. Additionally, we hoped to strengthen basic medical research and patient-centered care through feedback from patients affected with Alzheimer's disease (AD). Recent studies in model systems have shed light on genetic and epigenetic mechanisms of neurodegenerative diseases, including AD, Parkinson's related dementia, dementia with Lewy bodies, and frontotemporal dementia, among others. However, most studies have focused on establishing a solid knowledge base and may have missed important aspects of communications between patients with cognitive impairment and healthcare professionals. In addition, scientists are often out of the loop from such communication, which requires multiple steps before it becomes available to researchers and may be a source of unexpected bias. Thus, we propose to include scientists, especially those studying early-stage patients, in these communications so that patient input can be directly integrated into research protocols. In this manner, we hope to make a significant contribution by integrating basic medical sciences and patient-centered care.

Stigma can be defined as an unfavorable way of assessing a particular person or population with a misunderstanding (a knowledge problem), which may eventually result in prejudice (an attitude problem) and discrimination (a behavior problem) (Batsch and Mittleman, 2012). It is surprising to learn about misconceptions surrounding dementia and those who have cognitive impairment similar to those in AD. In the World Alzheimer Report 2012, Batsch and Mittleman (2012) state:

“Symptoms of dementia are perceived differently… considering dementia as a normal part of ageing, mental illness, something metaphysical linked to supernatural or spiritual beliefs… It is very important that there is better public awareness and understanding to reduce the stigma associated with dementia.”

How can the basic sciences help to reduce the stigma and embarrassment that are often associated with dementia? The American Alzheimer's Association has initiated a vital program that invites patients, caregivers, and advocates to the National Alzheimer's Association conference to be held in the summer of 2014 (http://www.alz.org/). The Society of Neurosciences also has an outreach program (http://www.sfn.org/), though it focuses on exposing young students to neuroscience research. Although these resources provide opportunities for research education and interactions among early-stage patients, clinicians, and caregivers, there are still limited opportunities to facilitate effective communications with scientists.

## Outreach to and from basic medical researchers to patients

### Three key factors

In this section, we discuss the following key factors that may help build specific strategies, initiatives, and programs: (1) better education using clear verbal and written communication with early-stage patients with cognitive impairments, (2) better involvement of early-stage patients, using a precise protocol to record their feedbacks, and (3) participation of medical students, scientific researchers, and healthcare providers, before or early in their studies, to understand what patients are experiencing, therefore allowing research to better target the problems that these patients express.

First, although talking to patients is often not part of scientific studies, it can be highly informative for scientists to be part of the communication between patients with cognitive impairment and healthcare professionals. We believe it is important that scientists carefully listen to early- and more advanced stage patients. Some patients can explicitly explain their experiences and feelings with regard to their memory impairment. Thus, researchers and healthcare professionals can better understand the thoughts of those who are impaired. Early-stage patients have enough cognition to clearly explain their individual situations, which may differ from patient to patient. Generally, they have the capability to verbalize much of their moment to moment activities during the day, in terms of what they can and cannot do. Here are some examples:

1. A patient stated, “I have problems with writing e-mails or any letters, but I can write with greater ease when I am listening to music.” The conversation with the patient was spontaneous and routine, but his ability to write while listening to music delivered an important message to one of the authors; the effects of music can be studied in the laboratories of model systems. Music is known to reduce anxiety and is dependent on ovarian steroid hormones, including progesterone, in female mice (Chikahisa et al., 2007). Music is also known to modulate brain-derived neurotrophic factor, which is essential for neuron survival and function (Li et al., 2010). In addition, the effects of music may be studied using “omics” analysis in model systems with auditory functions, including fish and rodents.
2. Another conversation mentioned that some patients with AD had trouble with falling, which inspired Machino et al. (2014) to revisit locomotion analysis of a *C. elegans* strain over-expressing beta amyloid (referred to as AD strain). This AD strain has been reported to show relatively normal locomotion (Dosanjh et al., 2010). However, the study used a manual assay and may have missed locomotion deficits. Using a semi-automated assay with better accuracy, they found that the AD strain shows reduced locomotion speed (Machino et al., 2014). Importantly, motor activities are not always defective in AD but are abnormal (Webster et al., 2014). Thus, studies with model systems could possibly identify how behavioral disturbances may occur in AD.
3. AD patients are advised to exercise, which may improve the quality of life or even delay functional declines (Press and Alexander, 2014). Exercise can be easily integrated into studies of locomotion and motor activity in model systems of AD (Dao et al., 2013; Intlekofer and Cotman, 2013).
4. Some patients experienced worsening symptoms in the late afternoon and evening. This symptom, called “sundawning,” is easily noticed when communicating with patients. The mechanism is not well understood but could be studied in mice (Bedrosian and Nelson, 2013).

It is essential to study sensitivity to AD and degeneration of neurons. Having relaxed and detailed conversations with early-stage patients could provide a number of potential approaches. Such communication would otherwise remain vague and unnoticed unless patient feedback is documented and easily accessible to scientists. Integration of research with patient feedback should be informative. Thus, a potential strategy could include: (1) opportunities to have routine communication, (2) documentation of potentially important information, and (3) incorporation of the information into research protocols. To integrate feedback into studies of model systems requires an additional step. As challenging as it may seem, it is our opinion that such feedback will eventually provide specific approaches to the study of AD by focusing on AD-resistant functions, as discussed below.

Second, communication with early-stage patients could better emphasize research that is oriented to the needs of the patients. A benefit of communication with early-stage patients is the ability to differentiate between what scientists seek (i.e., an understanding of mechanisms) and what patients need (i.e., treatment and an improved quality of life). Current research emphasizes the mechanisms causing AD and develops solutions to prevent AD. In contrast, patients need to have proper examinations, diagnoses, treatment plans, and a variety of lifestyle changes during the disease process, which unfortunately at this time has no cure. It is evident that there is a distinct gap between the needs of each patient and what some research may be providing—therapies that may be partially effective in terms of prevention and/or treatment. For this reason, scientists should reach out to patients to acquire feedback necessary to aid other researchers and healthcare providers in developing patient-centered care and research. Each patient is clearly different but, collectively, the information obtained may be highly beneficial.

Third, to close the knowledge gaps that likely exist, medical students, research scientists, and healthcare providers must have the best information from each patient. Grants and scientific meetings will also benefit from input by AD patients. To make these activities most effective for patient treatment, it is essential to have students spend more time with patients in a variety of outreach settings. In addition to the activities previously discussed (Batsch and Mittleman, 2012), there are pilot approaches being initiated. These include an international Alzheimer's association conference that will be held in the summer of 2014 and an outreach program where students can communicate their research to patients (Biotech Academy Touro Internship Program). Because there is presently no such process regarding outreach from model systems to patients (PubMed search using two key words, “model system” and “outreach”; accessed on March 28, 2014), this is most likely the first article that describes an outreach from model systems to patients.

## Outreach from model systems to knowledge

### Two types of resistance to AD

Among the many approaches in studying AD, we will review an approach that utilizes neurons and nerve function resistant to AD. Although a number of studies have focused on the disease, it is important to understand the well-preserved memory functions in dementia patients. The objective of this approach is to complement, activate, or preserve declining cognitive functions that are relatively resistant to dementia. They include cognitive exercises (Woods et al., 2012), music, and emotions (Baird and Samson, 2009; Groussard et al., 2013; Moore, 2013; Ueda et al., 2013). Physical exercise may also be included in this category. Implicit memory for music, including the ability to play instruments (procedural memory), is well-preserved in the middle to late stages of AD, whereas explicit memory is sensitive to AD in the early stages (Baird and Samson, 2009; Groussard et al., 2013). In addition, music may be beneficial in controlling emotion and memory (Moore, 2013; Ueda et al., 2013).

Despite the past belief that implicit memory is well-preserved, it is now accepted that certain types of implicit memory, including “old memory” and memory of well-learned training, are well-preserved during normal aging (Johnson et al., 2002; Howard and Howard, 2013; Murakami, 2013; Ward et al., 2013). Implicit memory includes associative learning and memory, which is well-conserved in model systems including nematodes (*C. elegans*) (Murakami, 2007, 2013; Stein and Murphy, 2012; Chen et al., 2013; Gkikas et al., 2014), flies (Yamazaki et al., 2010), bees (Behrends et al., 2007; Farooqui, 2014), snails (Hermann et al., 2007), rodents (Villarreal et al., 2004; Sharma et al., 2010; Woodruff-Pak et al., 2010), and humans (Labar et al., 2004) (summarized in an editorial, to be published). Nematodes show relatively well-preserved associative memory through food conditioning but show impairment in associative memory through conditioning to a lack of food (Murakami, 2007; Stein and Murphy, 2012; Chen et al., 2013; Murakami, 2013; Gkikas et al., 2014). In this regard, memory for survival and prosperity is presumably important for natural selection, which has been involved in the evolution of animal species.

## Concluding remarks

Aging brings vulnerability that occurs unexpectedly in middle life and is described as the “middle-life crisis theory” (Murakami et al., 2011; Murakami, 2013). This theory states that age-related changes are mostly reversible in the early phase and eventually transition to a more advanced and irreversible phase around the middle of the lifespan. The early phase of age-related memory impairment appears reversible and can be stored, for example, in bees (Baker et al., 2012) and *C. elegans* (Murakami et al., 2008; Stein and Murphy, 2012). According to this theory, the early phase involves a shift from one state to another rather than damage to biological functions. By contrast, dementia presumably involves mixed functions in neurons, including damaged (irreversible), less damaged/affected (reversible), and normal (well-preserved) functions. Understanding altered nerve functions as well as the interactions among functions should provide strategies for developing treatment options for dementia. Information resulting from research in model systems could lead to possible treatments that may stimulate or complement declining cognitive functions in dementia patients. Collaboration among scientists, healthcare professionals, and caregivers and listening to feedback from patients with early-stage AD and other types of dementia can hopefully lead to improvements in model systems and basic medical research.

### Conflict of interest statement

The authors declare that the research was conducted in the absence of any commercial or financial relationships that could be construed as a potential conflict of interest.

## Abbreviations

AD: Alzheimer's disease
AMI: age-related memory impairment.
